# Comments on: Isolated Nonocompaction Suggests Subclinical Myopathy

**DOI:** 10.1055/s-0038-1676600

**Published:** 2019-02

**Authors:** Josef Finsterer

**Affiliations:** 1 Krankenanstalt Rudolfstiftung, Messerli Institute, Veterinary University of Vienna, Vienna, Austria

It is with interest that I read the article by Nappi et al^1^ about a fetus with left ventricular hypertrabeculation (LVHT), also known as left ventricular noncompaction (LVNC), at 26 weeks and 4 days of gestation. The fetus was delivered by cesarean section, preterm, at gestational week 31 and died 3 days after delivery for unknown reasons. The autopsy did not reveal any cardiac abnormality in addition to LVHT.^1^ Thus, I have a few comments and concerns.

Since LVHT is frequently associated with monogenic disorders or chromosomal defects,^2^ it would be interesting to know if the fetus underwent genetic testing and if any previously described mutation or chromosomal abnormality associated with LVHT was detected. In case a genetic defect was detected, I would like to know if the parents were tested for this particular defect and if it could be found in either of the parents as well.

Since LVHT is particularly associated with neuromuscular disorders in up to 80% of the cases,^3^ it would also be interesting to know if muscle tissue of the fetus was collected to perform immunohistological, ultrastructural, or biochemical investigations. Neuromuscular disorders may present with only mild manifestations or may remain subclinical; thus, it would be interesting to know if creatine-kinase or serum lactate was elevated in the index patient or his parents.

I do not agree that LVHT is per definition associated with hypoplastic papillary muscles. Usually, papillary muscles are well preserved in patients with LVHT, and there is no indication for hypoplpasia or atrophy of papillary muscles. Papillary muscles may rather give rise to be mixed up with LVHT, particularly on axial views.^4^ Only in case LVHT is non-isolated, other cardiac abnormalities, including hypoplasia of the papillary muscles, may be present.^5^

A frequent complication of LVHT not mentioned in the article is ventricular arrhythmias. Ventricular arrhythmias may be the cause of sudden cardiac death. However, if ventricular arrhythmias are detected prior to a fatal outcome, the patient may survive upon adequate therapy. This includes antiarrhythmic medication or implantation of an implantable cardioverter defibrillator. Therefore, it is crucial to mention ventricular arrhythmias, as they are accessible to treatment if detected in due time. Ventricular arrhythmias may have been the cause of death of the the index patient 3 days after delivery.

A further shortcoming of the study is that it is not mentioned that LVHT may not be congenital in all cases. Although LVHT is congenital in the majority of the cases, it may be also acquired, thus developing after birth. Conditions associated with acquired LVHT include neuromuscular disorders, athletism, or pregnancy. Gestational LVHT in a pregnant female may disappear after delivery. Thus, transient LVHT may have a better prognosis than permanent LVHT.

In summary, it is crucial to investigate fetuses with LVHT genetically, to screen patients with LVHT for ventricular arrhythmias, and to investigate patients with LVHT for neuromuscular disorders. Acquired LVHT should be discussed, since it may have implications the outcome of these patients.

## Notes

Auhtors' Reply
**Luigi Nappi^1^ Lorenzo Vasciaveo^1^ Felice Sorrentino^1^ Gennaro Scutiero^2^ Piergiorgio Iannone^2^ Pantaleo Greco^2^**
^1^Department of Medical and Surgical Sciences, Institute of Obstetrics and Gynecology, Università di Foggia, Foggia, Italy ^2^Section of Obstetrics and Gynecology, Department of Morphology, Surgery and Experimental Medicine, Università degli Studi di Ferrara, Ferrara, Italy **Address for Correspondence**: Piergiorgio Iannone, MD, Instituto di Ginecologia ed Obstetricia, Dipartimento Di Morfologia, Chirurgia e Medicina Sperimentale, Università degli Studi di Ferrara, Azienda, Ospedaliero-Universitaria S. Anna, Via Aldo Moro, 8. 44121, Cona, Ferrara, Italy (e-mail: pg.iannone88@gmail.com). Dear Editor, We would like to thank the authors for their interest in our work titled “Fetal Noncompaction Cardiomyopathy and Histologic Diagnosis of Spongy Myocardium: Case Report and Review of the Literature”.^1^ We report a case of antenatal diagnosis of LVNC with no other cardiac or extracardiac anomalies at 26 weeks of gestation. We have read their considerations and offer responses to every point of the letter in order to enhance both the scientific value and clarity of the manuscript. The authors asked about any genetic testing perfomed. As already mentioned in our paper, the fetus underwent only a classic and not molecular karyotype study that was normal.^1^
The autopsy revealed that death was due to heart failure and the histological diagnosis was: “left ventricular heart wall characterized by accentuated muscular trabeculation and deep recesses affecting half of the entire thickness”, consistent with the sponge myocardium framework. No histological, immunological or biochemical studies on the fetal muscle tissue were performed in order to evaluate the association with neuromuscular disorders. Focusing on the parents, as already described in our article,^1^ they performed only an echocardiography that was normal. The family history was negative for cardiomyopathies. Moreover, we can add that in a subsequent pregnancy, the parents underwent genetic testing (array comparative genomic hybridization [aCGH]) and their karyotype was compared with the fetal one (an amniocentesis was performed studying the classic and molecular karyotype as a result of a prenatally diagnosed central nervous system [CNS] malformation). The result was negative both parents and fetus. With regards to the author's comment on the association with hypoplastic papillary muscles, we respectfully disagree. In an autopsy study of noncompaction in fetuses and neonates by Ursell,^2^ pathologic criteria for noncompaction included hypoplastic left ventricular papillary muscles, abnormal trabecular architecture and greater than 50% penetration of the left ventricular wall thickness by intertrabecular recesses. As reported in our work,^1^ the clinical presentation of LVNC varies from no symptoms to heart failure, embolism, arrhythmia, mitral insufficiency, conduction disorders and sudden death. We absolutely agree that ventricular arrhythmias may be the cause of sudden death; however, the fetus never presented anomalies of frequency arrhythmias. He was born alive and died after a few days due to heart failure. We do agree that LVHT can be congenital or acquired. In fact, as discussed in our study, patients with LVNC should be submitted to screening for congenital cardiac defects, genetic anomalies, and neuromuscular and metabolic diseases.^1^
We greatly appreciate the author's final considerations. However, we want to highlight that our case is based on an antenatal diagnosis of LVNC that was consecutive to the finding of cardiomegaly determined by the altered relationship between the thoracic circumference and the cardiac circumference and the consequent alteration of the cardiac contractility with hypomobilty. Ultrasound technical improvements with the development of new tools for the evaluation of the systolic and diastolic fetal function and advances in fetal cardiology will allow a risk stratification with a better evaluation of the prognosis of newborns with congenital heart disease and new and promising therapies for fetuses with cardiovascular disease.^3^
